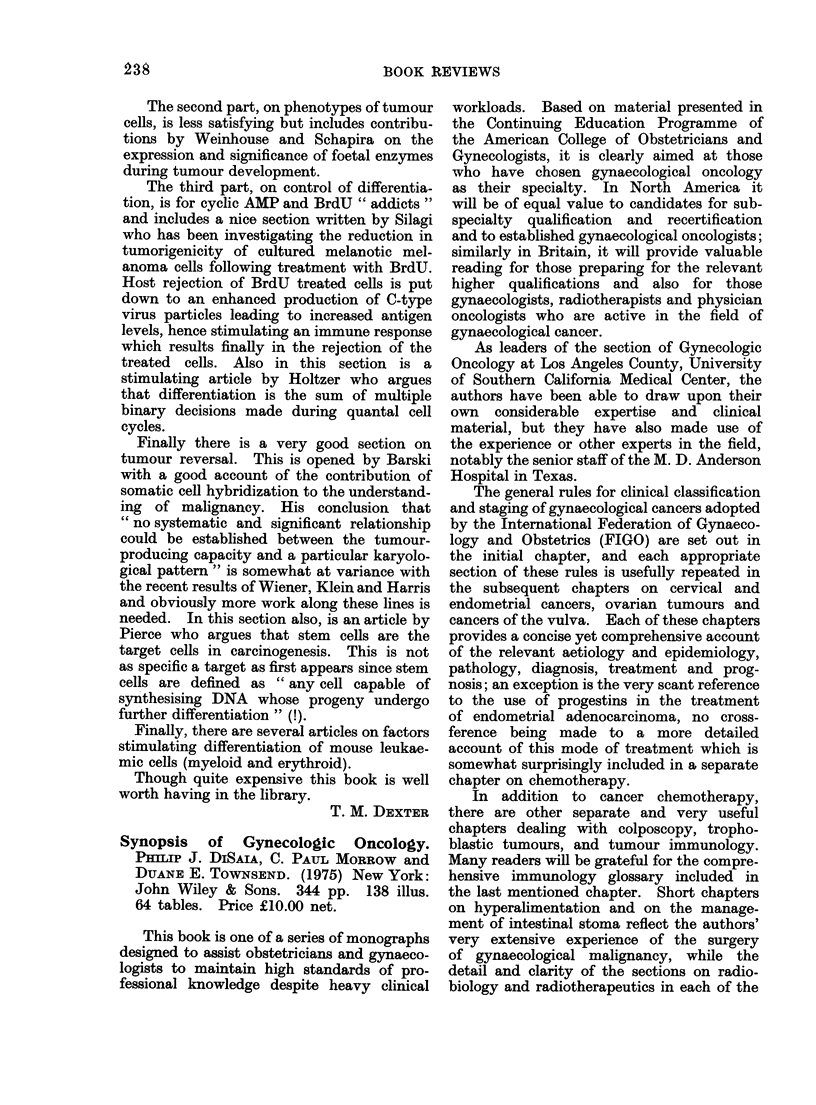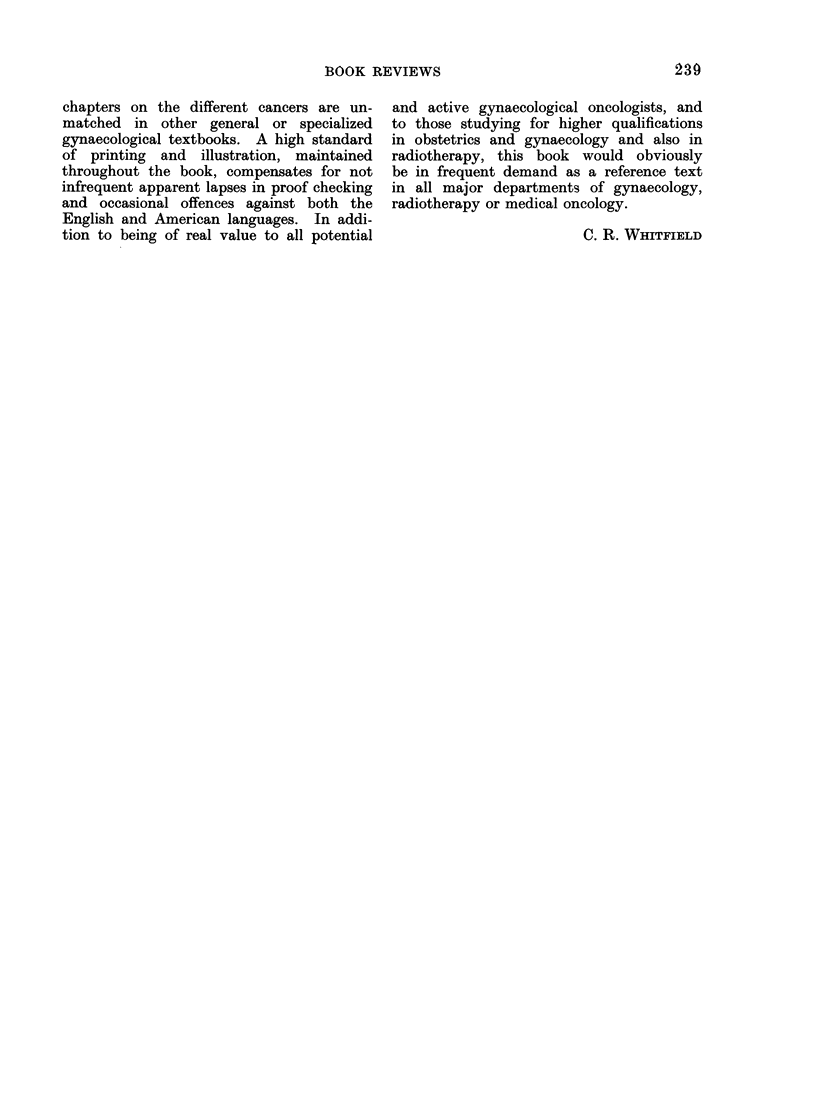# Synopsis of Gynecologic Oncology

**Published:** 1976-02

**Authors:** C. R. Whitfield


**Synopsis of Gynecologic Oncology.** Philip J. DiSaia, C. Paul Morrow and Duane E. Townsend. (1975) New York: John Wiley & Sons. 344 pp. 138 illus. 64 tables. Price £10.00 net.

This book is one of a series of monographs designed to assist obstetricians and gynaecologists to maintain high standards of professional knowledge despite heavy clinical workloads. Based on material presented in the Continuing Education Programme of the American College of Obstetricians and Gynecologists, it is clearly aimed at those who have chosen gynaecological oncology as their specialty. In North America it will be of equal value to candidates for subspecialty qualification and recertification and to established gynaecological oncologists; similarly in Britain, it will provide valuable reading for those preparing for the relevant higher qualifications and also for those gynaecologists, radiotherapists and physician oncologists who are active in the field of gynaecological cancer.

As leaders of the section of Gynecologic Oncology at Los Angeles County, University of Southern California Medical Center, the authors have been able to draw upon their own considerable expertise and clinical material, but they have also made use of the experience or other experts in the field, notably the senior staff of the M. D. Anderson Hospital in Texas.

The general rules for clinical classification and staging of gynaecological cancers adopted by the International Federation of Gynaecology and Obstetrics (FIGO) are set out in the initial chapter, and each appropriate section of these rules is usefully repeated in the subsequent chapters on cervical and endometrial cancers, ovarian tumours and cancers of the vulva. Each of these chapters provides a concise yet comprehensive account of the relevant aetiology and epidemiology, pathology, diagnosis, treatment and prognosis; an exception is the very scant reference to the use of progestins in the treatment of endometrial adenocarcinoma, no crossference being made to a more detailed account of this mode of treatment which is somewhat surprisingly included in a separate chapter on chemotherapy.

In addition to cancer chemotherapy, there are other separate and very useful chapters dealing with colposcopy, trophoblastic tumours, and tumour immunology. Many readers will be grateful for the comprehensive immunology glossary included in the last mentioned chapter. Short chapters on hyperalimentation and on the management of intestinal stoma reflect the authors' very extensive experience of the surgery of gynaecological malignancy, while the detail and clarity of the sections on radiobiology and radiotherapeutics in each of the chapters on the different cancers are unmatched in other general or specialized gynaecological textbooks. A high standard of printing and illustration, maintained throughout the book, compensates for not infrequent apparent lapses in proof checking and occasional offences against both the English and American languages. In addition to being of real value to all potential and active gynaecological oncologists, and to those studying for higher qualifications in obstetrics and gynaecology and also in radiotherapy, this book would obviously be in frequent demand as a reference text in all major departments of gynaecology, radiotherapy or medical oncology.

C. R. Whitfield